# Sodium-Glucose Cotransporter-2 Inhibitor-Induced Euglycemic Diabetic Ketoacidosis in a Type 2 Diabetic Patient

**DOI:** 10.7759/cureus.51184

**Published:** 2023-12-27

**Authors:** Rakahn Haddadin, Danny Aboujamra, Homayon Iraninezhad

**Affiliations:** 1 Medicine, MountainView Hospital, Las Vegas, USA; 2 Internal Medicine, St. George’s University School of Medicine, Las Vegas, USA; 3 Gastroenterology, MountainView Hospital, Las Vegas, USA

**Keywords:** gastroenterology hepatology, sodium-glucose cotransporter-2 (sglt-2) inhibitors, adult gastroenterology, general internal medicine, medication-induced pancreatitis

## Abstract

Euglycemic diabetic ketoacidosis (euDKA) is a life-threatening metabolic complication typically associated with type 1 diabetes mellitus (T1DM). However, its occurrence in type 2 diabetes mellitus (T2DM) remains exceptionally rare. We present a case report detailing the unusual manifestation of euDKA in a patient with T2DM following the initiation of treatment with a sodium-glucose cotransporter-2 (SGLT-2) inhibitor. The patient, a 67-year-old female with a history of T2DM and well-controlled blood glucose levels, was commenced on an SGLT-2 inhibitor as part of her antidiabetic regimen just two weeks prior. Subsequently, the patient developed euDKA despite maintaining near-normal glycemic levels. This paradoxical presentation challenges the conventional understanding of DKA in T2DM and underscores the need for heightened clinical awareness. EuDKA associated with SGLT-2 inhibitors is an infrequently reported phenomenon, further complicating the clinical landscape. This case contributes to the growing evidence suggesting an association between SGLT-2 inhibitors and the development of euDKA in patients with T2DM. The rarity of this occurrence necessitates a thorough exploration of potential risk factors and underlying mechanisms.

## Introduction

Diabetic ketoacidosis (DKA) is known as a life-threatening acute complication of type 1 diabetes mellitus (T1DM). DKA mainly occurs in patients with T1DM; however, it can occur in T2DM if there is any acute trigger such as an infection, trauma, or acute coronary syndrome [1]. The diagnosis of DKA is confirmed by the presence of several criteria: blood glucose greater than 250 mg/dL, pH below 7.3, serum bicarbonate less than 18 mEq/L, presence of high urinary or blood ketones, and an elevated anion gap metabolic acidosis [2,3]. 

There is, however, a manifestation where an elevated anion gap metabolic acidosis is present with ketonemia/ketonuria and blood glucose levels less than 250 mg/dL [4]. These criteria illustrate the life-threatening disease known as euglycemic DKA (euDKA). The incidence of euDKA has grown with the introduction and increasing use of SGLT-2 inhibitors in the market [5]. In this report, we describe a case of euDKA developing in a type 2 diabetic patient on empagliflozin, presenting with acute pancreatitis. 

## Case presentation

A 67-year-old female with a past medical history of T2DM, hypertension (HTN), dyslipidemia, and atrial fibrillation on apixaban for anticoagulation presented to the hospital for intractable abdominal pain, shortness of breath, and chest pain. She reported worsening abdominal pain over the last few days, along with nausea and reported episodes of vomiting. She had previously been discharged from another hospital for similar symptoms after imaging and diagnostic workups were all negative. 

Upon admission, the patient’s vitals were unremarkable, and she did not require supplemental oxygen. Initial labs showed a complete blood count that was unremarkable. In contrast, the chemistry panel in Table 1 was significant for a bicarbonate of 15 mmol/L, anion gap of 24 mmol/L, and glucose of 188 mg/dL. The urinalysis results shown in Table 2 were significant for large ketones, glucose at 1000 mg/dL, trace protein, and small leukocyte esterase. 

**Table 1:** Chemistry panel. BUN: blood urea nitrogen.

Comprehensive metabolic panel	Results (reference)
Sodium	138 mmol/L (135-145)
Potassium	4.2 mmol/L (3.5-5.5)
Chloride	89 mmol/L (93-107)
Carbon dioxide	15 mmol/L (21-32)
Anion gap	24 mmol/L (4-12)
BUN	26 mg/dL (7-18)
Creatinine	0.81 mg/dL (0.52-1.23)
Albumin	2.6 g/dL (6.4-8.3)
Lactic acid	3.5 mmol/L (0.4-2.0)
Glucose	188 mg/dL (70-110)
Hemoglobin A1c	10.2 % (4.2-6.3)

**Table 2:** Urinalysis.

Urinalysis	Results (normal reference)
Urine color	Yellow (yellow)
Urine appearance	Cloudy (clear)
Urine pH	5.5 (5.0-9.0)
Urine protein	Trace mg/dL (negative)
Urine nitrites	Negative (negative)
Urine leukocyte esterase	Small (negative)
Urine glucose	1000 mg/dL
Urine ketones	Large

On admission, the patient described the pain as mid-epigastric with radiation to the back that worsens with positional movement. The patient experienced multiple episodes of nausea and vomiting, which worsened with drinking and eating. The patient's pain was relieved with a complete restriction of diet and liquids and intravenous morphine for pain medication (IV). 

The patient denied any alcohol use, previous history of gallstones, recent trauma, or autoimmune disease history. Reviewing the patient's home medication shows atenolol 50 mg, apixaban 5 mg, and, most recently, empagliflozin 25 mg daily, which the patient started two weeks before initial hospital presentation and symptom onset. 

**Figure 1:**
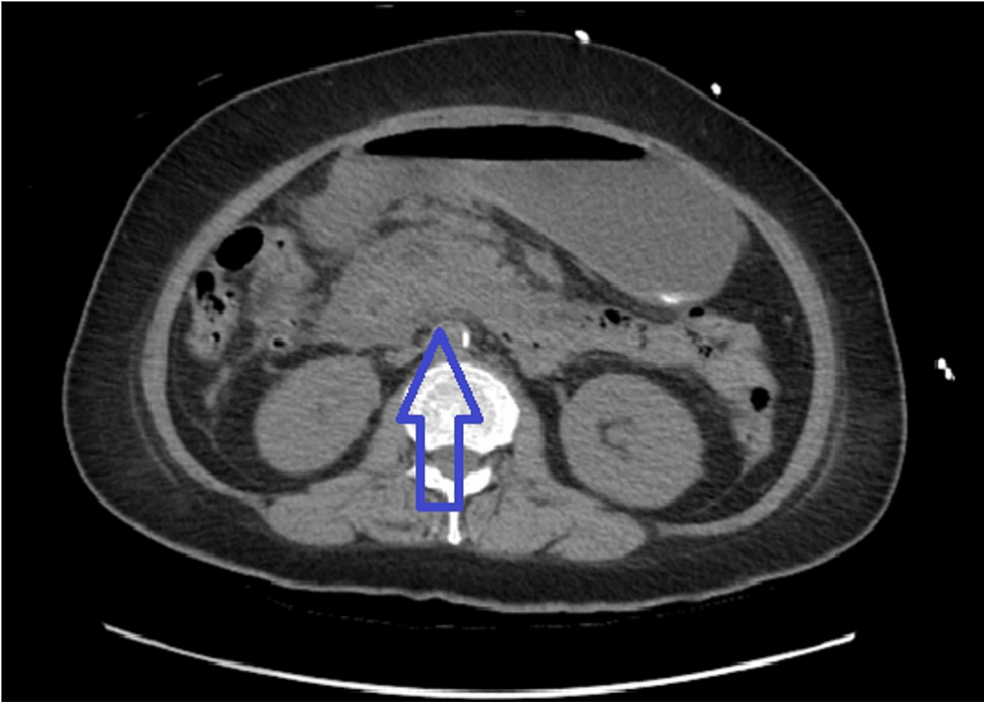
Increased edematous changes at the pancreas consistent with acute pancreatitis.

Physical examination was significant for severe tenderness to palpation in the mid-epigastric region. A computed tomography (CT) scan of the abdomen in Figure 1 shows increased edematous changes in the pancreas, consistent with acute pancreatitis.

The patient was admitted with a diagnosis of euDKA and acute pancreatitis and was started on conservative treatment with subcutaneous insulin, nothing by mouth, IV fluid, antiemetics, and a pain regimen that consisted of IV morphine. A magnetic resonance cholangiopancreatography (MRCP) done during hospitalization showed acute pancreatitis with a surrounding phlegmonous process with proteinaceous/hemorrhagic products. MRCP further identified no filling defects within the biliary system or intra- or extrahepatic biliary dilatation. 

Empagliflozin was discontinued upon diagnosis, and the patient was managed with supportive care, including being placed on a nothing-per-mouth protocol and intravenous fluids. The patient was started on a mild insulin sliding scale for glucose control, which she tolerated well. Table 3 shows the patient’s average daily glucose level readings during hospitalization.

**Table 3:** Blood glucose levels.

Blood glucose levels	Results (reference: 70-110 mg/dL)
Day 1 (day of admission)	188
Day 2	273
Day 3	150
Day 4	192
Day 5	179
Day 6	107
Day 7	111
Day 8	135
Day 9	151
Day 10	158
Day 11	166
Day 12	198
Day 13	207
Day 14	114
Day 15	131
Day 16	150
Day 17	139
Day 18	154

Upon discharge, we recommended that our patient discontinue the empagliflozin indefinitely and continue her atenolol and apixaban. Additionally, we advised her to follow up with an endocrinology clinic for continuous monitoring of T2DM and medication adjustment.

## Discussion

The clinical triad for DKA is known as hyperglycemia, ketosis, and anion gap metabolic acidosis, whereas definitions of euDKA include blood glucose <250 mg/dL in the presence of severe metabolic acidosis (arterial pH below 7.3, serum bicarbonate less than 18 mEq/L) and ketonemia [6]. EuDKA occurs in both T1DM and T2DM; however, it seems more common in patients with T1D, with a prevalence ranging from 4.6 to 8.0 per 1000 patient-years, making our case of euDKA in T2DM unique [7]. A case literature review found SGLT inhibitor-associated DKA in type 1 diabetics to occur at a rate of 5%-12%, compared with <0.1% in T2DM [8]. The temporal relationship between the patient’s empagliflozin initiation and the onset of euDKA strongly suggests a potential causal association. The rates of euDKA during clinical trials of SGLT2 inhibitors were 0.2-0.6 per 1000 patient-years for 10 mg and 25 mg of empagliflozin, respectively [6]. 

In early 2015, the US Food and Drug Administration (FDA) issued a warning regarding SGLT2 inhibitor-induced DKA, in which there had been 20 reported cases [9]. The median time to DKA after initiating SGLT2 inhibitor therapy was two weeks. Approximately 50% of cases were associated with precipitating events such as infection or surgery, reduced oral intake, and reduced insulin dose [10].

The mechanism of action of SGLT-2 inhibitors, such as empagliflozin, involves altering the kidney’s glucose reabsorption. Plasma glucose is freely filtered by the glomerulus and reabsorbed in the proximal renal tubule, with a small amount excreted directly in the urine. Reports show up to 90% of renal glucose reabsorption occurs in the proximal tubule due to SGLT-2 [11]. At maximal doses of SGLT-2 inhibitors, glucose reabsorption decreases to less than 50% of the filtered glucose load [12].

Although the development of euDKA after SGLT-2 inhibitor use is a subject of ongoing research, the proposed mechanism stems from decreased plasma glucose levels and insulin release [13]. There is an upregulation of lipolysis, ketogenesis, and glucagon that overcomes this carbohydrate deficit [11]. This results in a volume-depletion state, contributing to euDKA in the setting of relative insulinopenia and increased plasma catecholamine and corticosterone levels [14].

A common theme with the proposed risk factors for euDKA is that they influence a state of metabolic starvation. Examples of triggers reported preceding the development of euDKA in individuals include persistent vomiting, dehydration, anorexia [15], and prolonged fasting [16]. Cited literature shows glucagon and endogenous glucose levels increase after dapagliflozin use [17]. The upregulated glucagon activity caused by metabolic starvation may thus partly explain the development of euDKA in those patients concurrently using SGLT-2 inhibitors, as the increase in glucagon levels further influences ketogenesis and an increase in insulin resistance [18].

An algorithm for managing euDKA has been suggested to assist clinicians in preventing the delay in time for diagnosis and worsened outcomes. This algorithm is similar to DKA and involves monitoring anion gap and ketones to guide insulin and fluid management. Additionally, a gradual transition to subcutaneous insulin is recommended to prevent relapse [7]. Timely diagnosis is crucial for heightened awareness of euDKA, as early identification and treatment can enhance clinical outcomes.

Considering the increasing use of SGLT-2 inhibitors in cardiovascular and renal diseases, reviewing and determining the risk factors that can predispose to euDKA are essential. Patients should also be educated on the possibility of developing euDKA when taking SGLT-2 inhibitors and its presentation, such as nausea and vomiting, with regular at-home glucose readings.

Furthermore, the absence of hyperglycemia should not dissuade medical providers from initiating treatment when there is a high enough suspicion of euDKA. 

## Conclusions

The report emphasizes the importance of recognizing euDKA as a potential complication in patients with T2DM, particularly those prescribed SGLT-2 inhibitors. Clinicians should exercise vigilance in monitoring patients for ketosis, irrespective of their glycemic status. Furthermore, the scarcity of reported cases underscores the need for additional research to elucidate the specific mechanisms by which SGLT-2 inhibitors may induce euDKA in T2DM patients, aiding in the development of safer therapeutic strategies.

In conclusion, this case report highlights the uncommon occurrence of euDKA in T2DM, especially with SGLT-2 inhibitor therapy. Increased awareness, careful monitoring, and further research are imperative to enhance our understanding of the complex interplay between these factors, ultimately improving patient safety and optimizing diabetes management.
